# Coaxial capacitor (COCA) coil for stretchable arrays in ultrahigh-field MRI

**DOI:** 10.1063/5.0304790

**Published:** 2026-01-28

**Authors:** Ming Lu, Pingping Li, Jason E. Moore, Xiaoyu Jiang, John C. Gore, Xinqiang Yan

**Affiliations:** 1 Vanderbilt University Institute of Imaging Science, Vanderbilt University Medical Center, Nashville, Tennessee 37232, USA; 2 Philips, Nashville, Tennessee, USA; 3 Department of Radiology and Radiological Sciences, Vanderbilt University Medical Center, Nashville, Tennessee 37232, USA; 4 Department of Electrical and Computer Engineering, Vanderbilt University, Nashville, Tennessee 37232, USA

## Abstract

Stretchable RF coils offer the potential to improve MRI performance by conforming closely to patient anatomy, regardless of patient size, thereby enhancing both signal-to-noise ratio (SNR) and patient comfort. In this work, we investigate a stretchable receive array design based on the coaxial capacitor (COCA) coil for 7 T MRI, constructed primarily from ultra-flexible Litz wire stitched onto elastic fabric substrates. The COCA coil eliminates the need for lumped capacitors and maintains stable decoupling performance under transverse stretching, provided that the overlapped area and the coil area change proportionally as the coil is stretched. Bench tests and phantom imaging experiments demonstrate that elliptical COCA coils (in the non-stretched state) maintain consistent decoupling characteristics across stretch ratios up to ×1.3 and outperform fixed arrays in SNR across varying phantom sizes. The proposed design shows strong potential for integration into wearable coil arrays, enabling improved imaging quality and adaptability for diverse patient anatomies.

## I. INTRODUCTION

Size-adaptable radio frequency (RF) coils can conform closely to the target anatomy across a range of patient sizes, thereby maximizing the filling factor and improving the signal-to-noise ratio (SNR).^1,2^ A well-established strategy for achieving size adjustability involves mechanical designs integrated into rigid or semi-rigid housings. For example, Adriany *et al.* developed a geometrically adjustable microstrip transmission line array for 7 T head imaging, incorporating a tunable patch capacitor network between adjacent elements.^3^ This configuration allowed the decoupling capacitance to automatically adapt to changes in coil geometry. Zhang *et al.* introduced a trellis structure for cylindrical coil arrays,^4^ where a mechanical frame maintained a constant element overlap during expansion or contraction. Gruber *et al.* designed a coil with meandered conductors that could stretch in one direction while mounted on a semi-rigid frame.^5^ These designs enable repeatable and robust adjustments; however, their rigid or semi-rigid housings can limit the coil’s ability to conform to anatomies with pronounced curvature.

An alternative approach eliminates rigid housings entirely and focuses instead on stretchable materials and configurations.^6,7^ One such method utilizes liquid metal conductors encapsulated within stretchable channels, enabling adjustability in one direction while maintaining the other dimension.^6^ This technique offers excellent flexibility and adaptability, although it requires specialized sealing methods and fabrication procedures, which may increase manufacturing complexity. Another method involves stitching flexible conductors onto a stretchable fabric substrate. If the conductor is sufficiently flexible, the coil can be stretched along one axis, and the fabrication process remains relatively straightforward. However, this design inherently maintains a constant total conductor length, meaning that stretching in one direction reduces the coil’s dimension in the perpendicular direction.

This concept dates back to at least 2012, when Nordmeyer-Massner *et al.* stitched copper braid onto elastic fabric,^8^ creating a size-adjustable knee coil. More recently, conductive thread was used in place of copper braid to achieve similar stretchable properties.^9,10^ In stitched designs, lumped capacitors are typically employed as distributed capacitive elements when needed. These components help maintain a high unloaded quality (Q-) factor but require careful mechanical design to prevent failure of solid components and solder joints under repeated stretching and bending.

If the coil is sufficiently flexible and shape-adjustable, it can be made stretchable by stitching its conductor onto a stretchable fabric substrate. Recently, we proposed a coaxial capacitor coil (COCA) for 7 T MRI,^11^ constructed primarily from ultra-flexible Litz wire. This design does not require lumped components apart from those on the feed board. In this work, we explore the feasibility of adapting the COCA design for stretchable coil applications and demonstrate its suitability for 7 T MRI. Since the stretchable COCA coil employs the same conductor materials and coaxial capacitor structure as the previously reported design, its intrinsic Q-factor is expected to be comparable to that reported in Ref. 11. The inherent flexibility and distributed capacitance of the COCA structure offer promising advantages for conformal and size-adjustable coil configurations.

## II. METHODS

### A. Circuit diagram of a single COCA coil

**FIG. 1.**
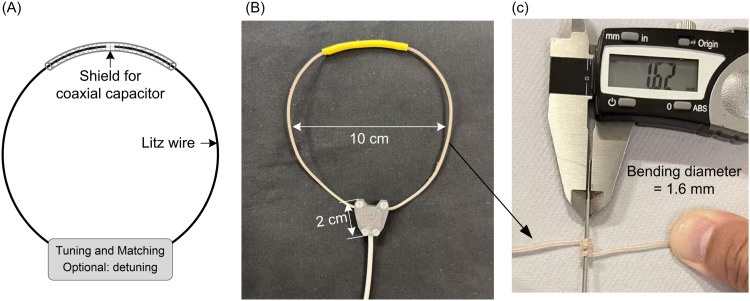
(A) Circuit diagram and (B) photograph of a single 10-cm-diameter coaxial capacitor coil designed for 7 T MRI. The exceptional flexibility of the Litz wire, which forms the majority of the coil, is demonstrated by its ability to bend around a rod as thin as 1.6 mm (C).

The circuit diagram of a 10-cm-diameter 7 T COCA coil is shown in Fig. 1(A). The coil has a total conductor length of ∼31 cm, with about 90% (27.5 cm) constructed from ultra-soft Litz wire. The wire has a diameter of 0.5 mm, and its polyethylene insulation adds an additional 0.5 mm in thickness. The feed board, which includes the tuning, matching, and detuning circuitry (for Rx-only), is housed in a “V”-shaped enclosure, with each side measuring ∼2 cm.

As previously discussed, flexibility is a critical attribute for such stretchable coil designs as excessive rigidity would compromise their ability to conform and stretch. The flexibility of the Litz wire is illustrated in Fig. 1(C), where it is shown tightly wound around a rod with a diameter of just 1.6 mm. It should be noted that a typical coaxial cable of similar size can only be wound around a rod with a minimum diameter of 7 mm. The exceptional flexibility of the COCA coil’s primary conductor makes it highly suitable for integration into stretchable coil designs. In the stretchable implementation, the entire coil, including the segmented coaxial capacitor region, is stitched onto a highly elastic fabric substrate such that mechanical deformation during stretching within the investigated stretch range is primarily absorbed by the fabric rather than the conductor itself.

### B. Stretchable COCA coil array with two elements (circular and elliptical in non-stretched state)

**FIG. 2.**
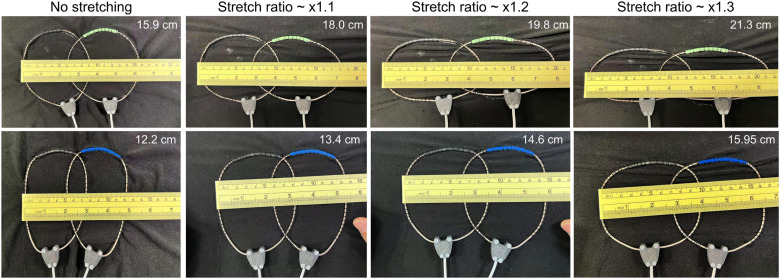
Photographs of two COCA coils stitched onto an elastic fabric, with the fabric and coils stretched at different stretch ratios in the transverse direction. Top row: Circular coils in the non-stretched state. Bottom row: Elliptical coils in the non-stretched state.

The top row of Fig. 2 presents a photograph of a two-element stretchable coil array. Each element is a 10-cm-diameter circular COCA coil in the non-stretched state. The coils were stitched onto an elastic fabric substrate (90% polyester and 10% spandex) to provide stretchability. The bottom row of Fig. 2 shows a photograph of another two-element stretchable coil array, in which each element is a COCA coil with a roughly elliptical shape instead of a circular shape in the non-stretched state. The long axis of the elliptical coil was designed to align with the z-direction (longitudinal direction in MRI), while the short axis aligned with the transverse direction. The long and short axes of the elliptical coils are ∼11.5 and 7.5 cm, respectively.

One challenge in stretchable coil design is preserving matching, tuning, and decoupling performance during deformation. To assess robustness, we conducted bench tests, evaluating these parameters as a function of stretching. Since the coil is typically designed to fit smaller anatomies snugly, it is often stretched in the transverse direction to accommodate larger circumferences. This makes transverse stretch the most common operating condition.

The scattering (*S*-) parameter (return loss and transmission coefficient) plots vs frequency were recorded as coils were stretched along the transverse direction by factors of ×1.1, ×1.2, and ×1.3. No retuning or rematching was performed when the coil was stretched. During the bench test, the coils were directly placed on a body-shaped phantom. The phantom has an octagonal shape—with a height of ∼25 cm, a width of ∼35 cm, and a length of ∼40 cm—a conductivity (*σ*) of 0.6 S/m, and a relative permittivity (*ɛ*_*r*_) of 78.

### C. Stretchable COCA coil array with five elements in volume configuration

**FIG. 3.**
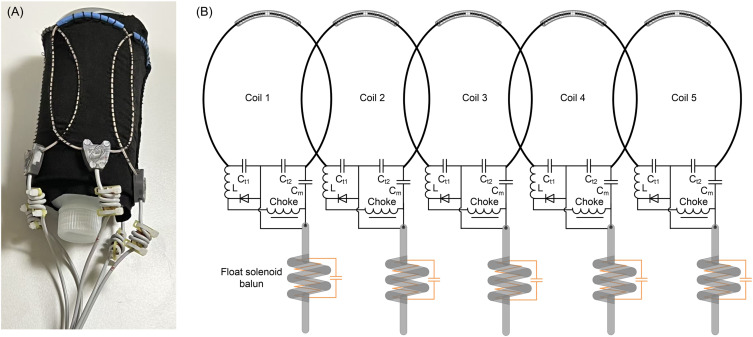
Photograph (A) and circuit diagram (B) of a five-channel stretchable COCA coil array designed for receive-only applications at 7 T.

Based on the bench test results (described in detail in the Results section), the elliptical-shaped coil was selected as the individual coil element for a volume-shaped stretchable array. A five-channel array with a non-stretched circumference of ∼20 cm was fabricated for 7 T MRI. Figure 3(A) shows the stretchable coil array wrapping around a 7.2-cm-diameter phantom. The array was implemented as a receive (Rx)-only design [Fig. 3(B)], intended for use in combination with the head volume transmit (Tx) coil (Nova Medical, USA). The stretchable array was connected to the Philips Rx interface box via non-magnetic RG174 cables, which carried both the RF signals from the coils and the DC signals from the interface. Cable lengths were carefully adjusted to achieve preamplifier decoupling, and floating solenoid baluns^12^ were incorporated to suppress unwanted common-mode currents. Adjacent elements were primarily decoupled through geometric overlap, with the measured coupling between neighboring coils better than −10 dB, and no additional decoupling networks were required.

### D. MRI experiments

To demonstrate the potential benefit of the stretchable coil, we measured the SNR on bottle phantoms with different diameters (6.2, 7.2, and 7.9 cm) on a 7 T whole-body scanner (Achieva 7 T scanner, Philips Healthcare, Best, Netherlands). All bottle phantoms were made from the same formula, containing 2.6 g/L NaCl and 1.25 g/L CuSO_4_. For comparison, SNR maps were also obtained using the same coil array mounted on a rigid housing with a fixed diameter of 8 cm. SNR maps were calculated based on low-flip-angle GRE images with the following parameters: FOV = 90 × 90 mm^2^, slice thickness = 2 mm, voxel size = 0.4 × 0.4 mm^2^, matrix = 160 × 160, TR/TE = 500/3.4 ms, flip angle = 20°, bandwidth = 445 Hz/pixel, and number of averages = 1.

In addition to the standard GRE imaging acquisition, noise-only maps were obtained using the same parameters, but with the RF transmit disabled. SNR maps were calculated using the Kellman method, which combines coil sensitivity profiles and noise covariance matrices to produce an optimal signal combination across channels.^13^

## III. RESULTS

### A. Bench test results

**FIG. 4.**
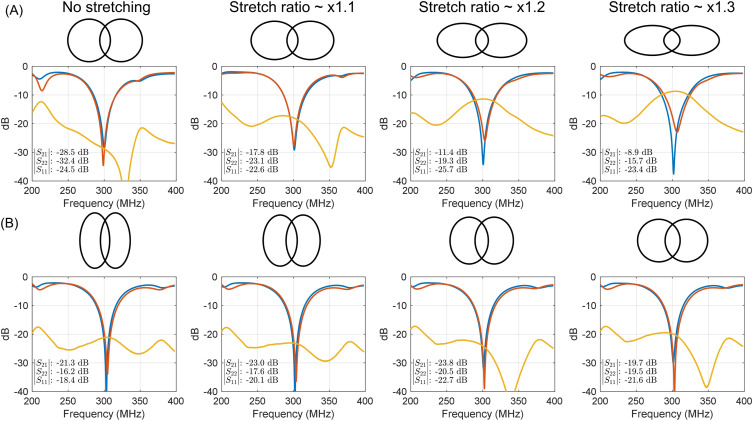
Measured S-parameter plots vs frequency for two coils stretched at different ratios along the transverse direction. (A) Circular coils in the non-stretched state. (B) Elliptical coils in the non-stretched state. Note that the coils were well-tuned and matched in the non-stretched state, and no retuning or rematching was performed when they were stretched.

Figure 4(A) presents the measured *S*-parameter plots (versus frequency) of two circular COCA coils in the non-stretched state and when uniaxially stretched by 10%, 20%, and 30% along the transverse direction. Figure 4(B) shows the *S*-parameter plots of two elliptical COCA coils in the non-stretched state and when stretched uniaxially along the transverse direction.

Across both initial geometries (circular and elliptical) and all stretch levels (×1.1–1.3), the input match consistently remains better than −15.7 dB. This robustness arises because the total conductor length (and thus the coil inductance) and the coaxial capacitance are essentially unchanged by stretching, resulting in no noticeable shift in resonance frequency. On the contrary, the coil-to-coil coupling behaves differently depending on the coil’s initial geometry. For the elliptical coils, coupling was almost unaffected by stretching, with *S*_21_ between adjacent coils changing only slightly from −19.7 to −23.8 dB [Fig. 4(B)]. In contrast, coupling between circular coils increased markedly when stretched, with *S*_21_ increasing from −28.5 to −8.9 dB [Fig. 4(A)].

It is well established that the degree of coupling between RF coils is highly sensitive to the ratio of the overlapped area to each coil’s enclosed area. Optimal decoupling is typically achieved when the overlap corresponds to ∼10% of each coil’s enclosed area. For circular coils, stretching into an elliptical shape reduces each coil’s own enclosed area but simultaneously increases the overlapped area (a vesica piscis shape). As a result, the overlap becomes larger than optimal, leading to notable increased coupling. In contrast, for elliptical coils, both the coil’s enclosed area and the overlapped area increase proportionally when stretched, keeping the overlap ratio nearly constant and thereby maintaining stable coupling. For this reason, elliptical coils in the non-stretched state were selected, owing to their robustness against stretch-induced coupling changes.

### B. Phantom MRI results

**FIG. 5.**
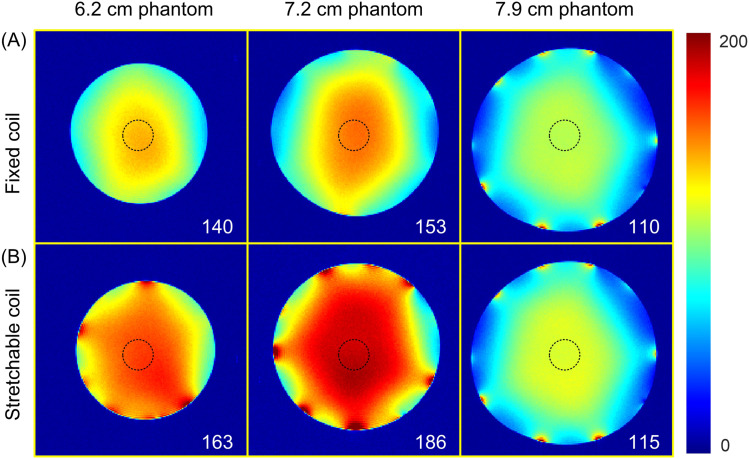
Measured multi-slice axial SNR maps on phantoms of different sizes, comparing coils mounted on a fixed housing to stretchable coils that can closely wrap around the phantom. (A) Fixed coil. (B) Stretchable coil.

Figures 5(A) and 5(B) display axial SNR maps acquired from different phantoms using the fixed and stretchable five-channel arrays, respectively. For the largest phantom (7.9 cm diameter), both the fixed and stretchable five-channel arrays exhibit high SNR. However, as the phantom size decreases—moving to medium and small phantoms—the fixed array shows reduced SNR compared to the stretchable array. This difference is most pronounced with the smallest phantom, where the central SNR of the fixed array drops to only 16% of that achieved by the stretchable coil. This outcome is expected as the fixed array remains farther from the phantom surface as size decreases, whereas the stretchable coil can consistently conform closely to the phantom, maximizing the receive sensitivity.

## IV. DISCUSSIONS AND CONCLUSION

This study demonstrates the feasibility of a stretchable COCA coil design for ultrahigh field MRI. The coil with elliptical geometry in non-stretched state maintains stable tuning and decoupling performance under transverse stretching and offers improved SNR across varying phantom sizes.

In this work, the coil was carefully arranged along the virtual ground plane, and a relatively remote floating solenoid balun^12^ was employed to minimize common-mode currents. While this approach proved effective, it should be noted that such a balun can become bulky when more coil elements are used, particularly in multi-row coil arrays. In future work, we plan to explore integrating a miniaturized balun circuit^14,15^ directly into the RF coil feed board to further suppress common-mode currents while preserving the coil’s stretchability and ergonomic advantages.

While the current prototype demonstrates improved imaging SNR along with excellent flexibility and stretchability, there is still room for further optimization. First, SNR could be further enhanced by increasing the coil’s unloaded Q-factor or reducing coil losses through the use of advanced materials. Second, to improve flexibility and stretchability, further miniaturization of the feed board could help reduce localized stiffness near the feed point. For example, the detuning circuit could be positioned remotely and integrated with the preamplifier board, reducing the number of components on the feed board. Third, the coil’s robustness against loading and transparency of Rx-only coils during the Tx period could be improved by using multiple coaxial capacitors in series.

## Data Availability

The data that support the findings of this study are available from the corresponding author upon reasonable request.
